# Can E-commerce development policies promote the high-quality development of agriculture?—A quasi-natural experiment based on a China’s E-commerce demonstration city

**DOI:** 10.1371/journal.pone.0299097

**Published:** 2024-05-29

**Authors:** Yaoguang Zhong, Fangfang Guo, Xi Wang, Junjun Guo

**Affiliations:** 1 School of Business, Dongguan Polytechnic, Dongguan, Guangdong, China; 2 Faculty of Humanities and Social Sciences, Macao Polytechnic University, Macau, China; 3 Business Administration Department, Guangzhou Open University, Guangzhou, China; 4 Institute of Public Policy, China West Normal University, Nanchong, Si Chuan, China; Harbin Institute of Technology, CHINA

## Abstract

As a new business model, E-commerce brings new changes to the global economy and society. So, can E-commerce development policies promote high-quality agricultural development? This article regards the pilot construction of national e-commerce demonstration cities as a quasi-natural experiment for the development of e-commerce. Based on the E-commerce pilot and economic and social development data of national prefecture-level cities from 2004 to 2018, the agricultural total factor productivity calculated by the Fare-Primont index method is used as the characterization of the quality of urban agricultural development, and an empirical model is constructed under the progressive Differences-in-Differences framework. This paper empirically tests the overall impact of E-commerce development on the high-quality development of agriculture and its sources, analyzes the heterogeneity and dynamics of the impact, and investigates the possible impact mechanism. The result shows that from the overall impact and its sources, the development of E-commerce in cities has a positive impact on the high-quality development of agriculture, and the impact is mainly due to its role in promoting technological innovation and economies of scale. From the perspective of heterogeneity, the larger the population size of a city, the more significant the level of e-conomic development or Internet development. From the perspective of the dynamic impact, the positive impact of e-commerce development becomes more significant over time. With the passage of time, the impact of E-commerce on high-quality agricultural development policies shows a trend of first increasing and then decreasing. Overall, this study provides empirical evidence for the rationality and effectiveness of policies and measures related to E-commerce to promote the high-quality development of agriculture.

## 1. Introduction

Governments around the world have permanently attached great importance to the development of agriculture. China is no exception and regards the development of agriculture as a fundamental national support policy. Different countries adopt different agricultural development models. For example, the United States adopts the large farm model, Japan chooses the precision agriculture model, France prefers the cooperative service model, and the Netherlands relies on the high-tech agriculture model. However, they all lack the integration of advanced industries or business models to drive high-quality agricultural development.

With the rise of E-commerce in recent years, countries worldwide have attempted to use E-commerce network markets to promote agriculture development and further expand agricultural product sales channels, such as Morocco [1] and Spain [2]. E-commerce plays a vital role in enhancing the vitality of economic development, improving the efficiency of resource allocation, promoting the development and upgrading of traditional industries, and opening up employment and entrepreneurship channels [3]. It has also become an essential driving force for China to cope with the economic downward trend and drive economic and social innovation and development [4]. In implementing the rural revitalization strategy, the role of E-commerce is also increasingly prominent. With the "industrial products to the countryside" and "agricultural products to the city", new E-commerce formats such as live broadcasts with goods, community group buying are emerging in rural areas [5]. China’s rural E-commerce online retail sales reached 2.17 trillion yuan in 2022, up 3.6 percent year on year, according to China’s Ministry of Commerce. The rapid development of E-commerce in rural areas not only benefits from the endogenous forces of the market, but also stems from government policy promotion [6]. The Chinese government selected 23, 30, and 17 "National E-commerce Demonstration Cities"(NEDCs) in 2011, 2014, and 2017, totaling 70 NEDCs. It aims to improve the industrial structure, promote economic development and upgrading, promote the development of E-commerce, especially the transformation of E-commerce in rural areas, and provide critical support for promoting agricultural modernization.

As a new economic form, the development of E-commerce can not only shorten the circulation link and save transaction costs, thus having multiple impacts on "agriculture, rural areas, and farmers", but also have a significant impact on agricultural and rural development by optimizing resource allocation and promoting technology spillover. Although the development of E-commerce helps to improve the quality of agricultural development, few existing studies directly examine the impact of E-commerce from the perspective of high-quality development. Even though few literatures focus on the relationship between E-commerce and agriculture development [7], they rarely provide reliable quantitative analysis and empirical evidence. So, how to objectively evaluate the effect of E-commerce development policy on high-quality agricultural development? How to verify whether the development of agricultural E-commerce policy has become an important driving force for high-quality agricultural development?

In view of this, in order to clarify the effect of improving the development quality of E-commerce, this study takes the Chinese government’s support for 70 NEDCs to develop E-commerce policies and promote agricultural economic development as a research perspective. This study takes the NEDCs as a quasi-natural experiment of urban E-commerce development policy, builds an empirical model under the dual difference framework, and empirically tests the impact of urban E-commerce development on high-quality agricultural development.

This study focuses on the following issues: (1) Whether and to what extent E-commerce development policy can promote the improvement of agricultural development quality; (2) Whether the impact of E-commerce development policy on high-quality agricultural development is different in regions with different primary conditions; (3) As time goes by, how will the impact of E-commerce development policy on farmers’ high-quality development change. The research will not only help us clarify the role of E-commerce in improving the quality of agricultural development and provide empirical evidence for the impact of E-commerce on agriculture, but also provide decision-making references and scientific advice for promoting high-quality agricultural development in rural revitalization.

This study is organized as follows: in Section 2, we present the literature review. In Section 3, we explain the empirical models and variables. Section 4 presents the results of the empirical study and the explanation of the results. We conclude this study in Section 5.

## 2. Literature review

This study mainly examines the impact of e-commerce development promotion policies on high-quality agricultural development. The literature closely related to the research topic of this paper is mainly developed from two aspects. First, the impact of e-commerce on agricultural development. The second is the measurement and influencing factors of high-quality agricultural development.

### 2.1 The impact of e-commerce on agricultural development

E-commerce can reduce information costs and promote economic development [8]. It is considered a practical solution to the urban-rural gap [9]. The sale of agricultural products through E-commerce improves the price transparency of agricultural products, reduces mediators, and improves market efficiency [10]. It has been widely used in developing countries such as India, Indonesia, and China, gradually expanding from large cities to small cities and villages [11]. The application of Internet of Things, big data and other technologies can not only provide more efficient, convenient and accurate information on the quality and safety of agricultural products for producers, sellers and ordinary users of agricultural products, but also improve the circulation efficiency of agricultural products and effectively reduce the circulation cost of agricultural products [12,13]. E-commerce has a strong radiation effect and spatial spillover effect. Through cluster effects, carbon emissions have been reduced [14,15], agricultural pollution has been alleviated [16,17], agricultural technological innovation has been promoted [18,19], and agricultural factor input efficiency has been improved [14]. The development of agricultural e-commerce will actively promote the upgrading and high-quality development of agricultural industry [4,20,21].

### 2.2 The measurement and influencing factors of high-quality agricultural development

Most scholars regard agricultural total factor productivity (ATFP) as a test indicator for high-quality agricultural development [22–24]. Scholars’ research on agricultural total factor productivity mostly focuses on regional difference analysis [25,26], spatial distribution [27,28], and the impact of different factors on agricultural productivity. Such as carbon trading [29,30], digital inclusive amount [31], environmental regulation [32], technological progress [33] etc. Many scholars have tested the influencing factors of high-quality agricultural development from different perspectives, for example, business environment [23], factor allocation [34], Ecological efficiency [24], market-oriented reform [35], agricultural digitalization [36,37], technological progress [33]. The method research of high-quality agricultural development generally adopts the location entropy index [38–40], panel vector autoregression (PVAR) model [23,34,40], Data envelopment analysis (DEA) [33], and SBM Undesired model [41].

The development of e-commerce has a positive impact on carbon emissions [42,43], agricultural ecological environment [16,17], and green innovation development [14,18]. However, due to the "endogenous" nature of E-commerce technology, existing research is mainly limited to its role in promoting the development of rural industries [4,44]. In the research on the influencing factors of high-quality agricultural development, e-commerce is rarely considered an essential factor. Regarding research methods, the DID double difference model is rarely used. Therefore, this study intends to conduct a direct empirical test of E-commerce’s "agriculture-related" impact from the perspective of high-quality development. In addition, to reduce the impact of “endogeneity” on the estimated results, this study also takes the construction of NEDCs as a quasi-natural experiment of urban E-commerce development. It constructs an empirical framework through a double difference model to realize the judgment of the causal relationship between E-commerce development and high-quality agricultural development. Thus, we can better understand the role and mechanism of e-commerce affecting agricultural development and provide strong support for promoting high-quality agricultural development.

This study has the following innovations. (1) In terms of research methods, this study uses the progressive differential model to study the impact of implementing of the NEDCs policy on the high-quality development of agriculture. Standard errors are clustered at the city level by simultaneously controlling for both time-fixed and city-fixed effects. While allowing systematic differences in the quality of agricultural development between cities, the autocorrelation effects within cities are controlled. (2) In terms of robustness test, this study examines the impact of national e-commerce demonstration city policies on high-quality agricultural development based on controlling national low-carbon pilot cities, carbon emission trading pilot cities, innovative pilot cities, and opening high-speed rail policies that affect high-quality economic development to make the test results more reliable. (3) In terms of estimation methods, considering the possible impact of selectivity bias and missing variable bias on the estimation results, this study adopts the PSM-DID method and instrumental variable method to estimate, so as to reflect whether the high-quality agricultural development in the region is affected before and after the pilot policy of NEDCs.

## 3. Theoretical framework

### 3.1 Theoretical analysis

Since the emergence of the new classical economic growth theory, mainly represented by the Solow model, total factor productivity has gradually been characterized by economists as the source of long-term sustained economic growth and the reference standard for evaluating the quality of economic development [23]. If the contribution rate of total factor productivity improvement dominates in the increment of total output of an economy, it is considered that the economy exhibits an intensive development model [24]. Namely, a high-quality development model. According to neoclassical economic theory, there are two main ways to improve total factor productivity: to increase production efficiency through technological progress and to improve resource allocation efficiency by recombining production factors [26].

**Fig 1:**
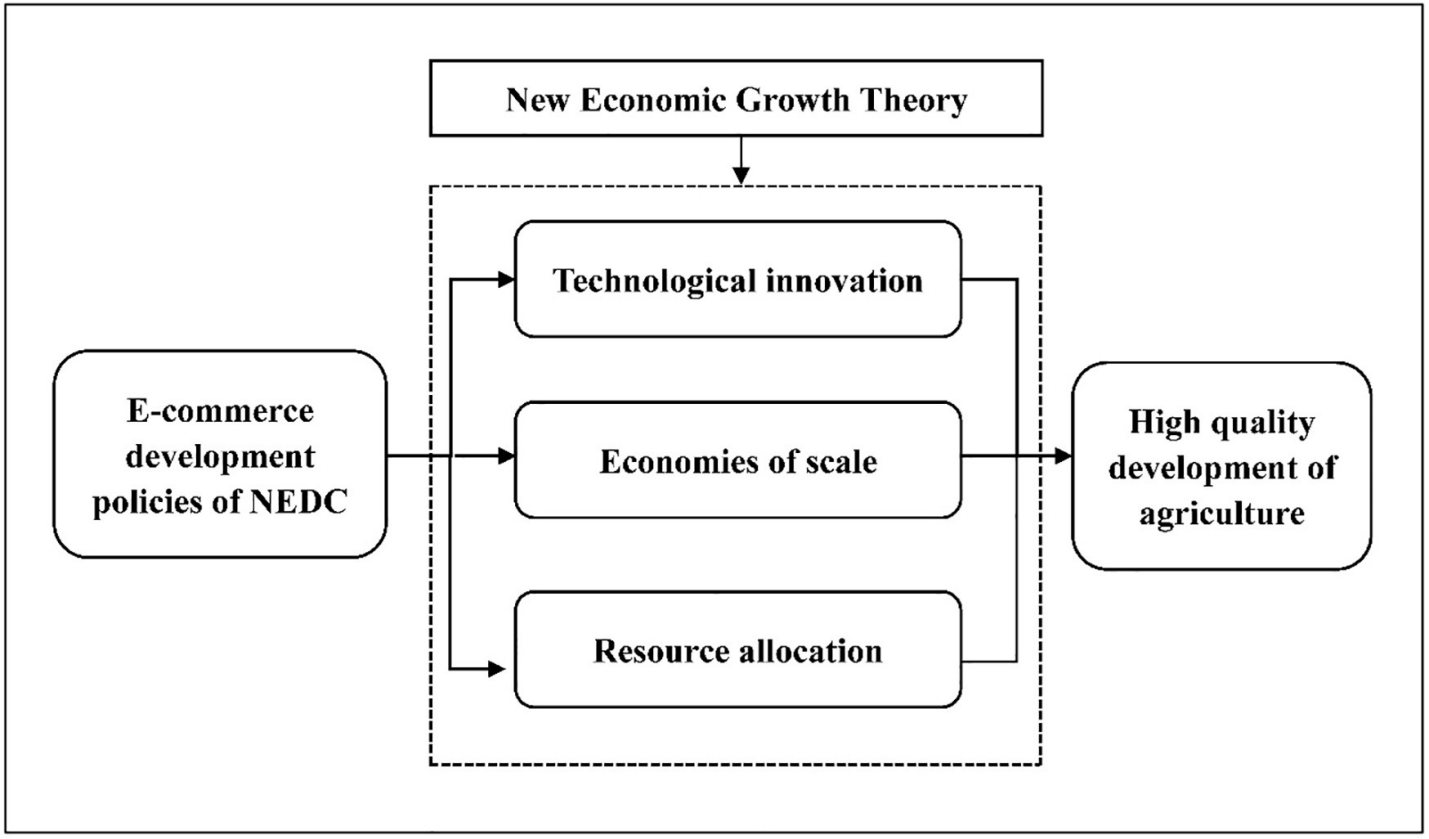
Theoretical model.

In agriculture, e-commerce is not limited by geography or time, which has changed the transaction mode of agricultural products. Online transactions have the advantages of fewer links, lower costs, and higher efficiency [10], attracting capital investment. Therefore, it promotes agricultural technological innovation [18], generates economies of scale [14], and promotes the effective allocation of agricultural resources [45]. We will examine the dynamic impact of NEDC’s e-commerce development policy on high-quality agricultural development from three aspects: technological innovation, economies of scale and resource allocation. According to the above analysis, this research theoretical model shown in Fig 1.

### 3.2 Dynamic impact mechanism

#### 3.2.1 Technological innovation

As the representative of modern information technology applications and the main form of the Internet economy, e-commerce will undoubtedly promote the digitalization level of "three rural areas" and agricultural technology innovation [46]. From the perspective of technological innovation, e-commerce is a product of high information technology, with the advantages of high efficiency, low cost, and real-time transparency across time and space constraints. The development of e-commerce contributes to the dissemination of technical knowledge and promotes the improvement of regional innovation levels and innovation environment [18]. Higher regional innovation levels and a better innovation environment will lead to faster agricultural technology progress, and agricultural technology progress is positively correlated with high-quality agricultural development.

Whether directly promoting the development of agricultural production technology or promoting the adoption of new technology through technology spillover, the promotion effect of e-commerce on agriculture-related technology shows dynamic characteristics. With the development of e-commerce, its technology upgrade potential is gradually released, the reform of agricultural technology is deepened, the scope of technology spillover is expanded, and agricultural technology innovation is accelerated [19]. After the gradual popularization of e-commerce-related agricultural technologies, the technological upgrading potential of e-commerce will be gradually released, and agricultural technology will not be significantly improved because of the development of e-commerce.

#### 3.2.2 Economies of scale

The rapid development of e-commerce can significantly break the boundaries between industries and promote cross-border integration, thus forming a whole industrial chain of clustering, informatization, and networking [29]. It effectively promoted the development of agricultural informatization, optimized the agricultural production mode and industrial chain, and aroused the enthusiasm of non-agricultural industry operators. Ultimately, by attracting capital investment, expanding the scale of agricultural production and operation, and reducing the operating costs of the industrial chain, economic benefits will be further improved, and the scale will be reflected. From the perspective of economies of scale, e-commerce has broken the geographical and time constraints and driven the development of upstream and downstream industries.

Whether it is the expansion of production and marketing scale or the expansion of the industrial chain, it is conducive to improving the quality of agricultural development [47]. The impact of e-commerce on agricultural economies of scale is mainly reflected in the improvement of internal and external coordination efficiency brought about by expanding the agricultural industry chain. Whether the collaborative efficiency can be improved is restricted by the optimal extension range. Before reaching the optimal range, with the development of e-commerce, the expansion of the agricultural industry chain will improve the coordination efficiency inside and outside the industry chain, which is manifested as economies of scale. However, after reaching the optimal range, the further expansion of the agricultural industry chain triggered by e-commerce will lead to the decline of agricultural economies of scale.

#### 3.2.3 Resource allocation

With the development of agriculture-related e-commerce, a large amount of capital and human capital will flow into the agricultural field. For the areas with great potential for agricultural resource development but a shortage of funds, the inflow of capital is conducive to the development and utilization of agricultural resources, effectively adjusting the ratio of capital and resources and optimizing the efficiency of resource allocation. From the perspective of resource allocation, the wide application and development of e-commerce will not only significantly improve the production and circulation efficiency of agricultural products improve the allocation efficiency of agricultural input factors but also bring about the improvement of the quality of agricultural development [48].

Driven by the potential high capital gains from the national e-commerce demonstration city construction policy, a large amount of material and human capital will flow into e-commerce-related fields. The development of the agricultural industry gradually leads to a surplus of capital, which reduces the efficiency of allocating agricultural resources and the available income of the agricultural industry. The decrease of agricultural industry income will drive away some capital and reduce resource misallocation, which may improve the efficiency of agricultural resource allocation.

From the above analysis, the impact of e-commerce on technological innovation, economies of scale, and resource allocation includes gradual strengthening factors and elements leading to attenuation. The influence of e-commerce on the high-quality development of agriculture results from the interaction of factors in different directions and will be dynamic with the effect of e-commerce.

Hypothesis 1: E-commerce development policies dynamically influence high-quality agricultural development through technological innovation.
Hypothesis 2: E-commerce development policies influence high-quality agricultural development through dynamic economies of scale.
Hypothesis 3: E-commerce development policies dynamically influence high-quality agricultural development through resource allocation.

## 4. Empirical model and variable selection

### 4.1. Empirical model

When testing the impact of E-commerce development on the high-quality development of agriculture, we can directly identify the high-quality development effect of E-commerce by regressing the agricultural development quality variable to the E-commerce development variable and according to the estimated value of the regression coefficient. However, E-commerce development is not exogenous for the high-quality development of agriculture. Its endogenous nature may come from two aspects. First, high-quality agricultural development has a reverse impact on E-commerce development. The higher quality and level of agricultural development provide soil for the development of E-commerce, which will also lead to a higher level of E-commerce development. Second, some missing variables that are difficult to measure, such as the development level of the Internet economy and the status of human capital, may affect the high-quality development of agriculture and E-commerce. When E-commerce development is not exogenous, a simple direct regression may lead to deviation in the estimation results due to "endogenous problems".

In order to reduce the impact caused by endogenous factors, we regard the construction of NEDCs as a quasi-natural experiment of local promotion of E-commerce development and set an empirical analysis model based on the dual difference framework. The double difference method is usually used to evaluate the impact of a policy or event on the implementation object and has been widely used at home and abroad [49,50]. The basic principle of this method is to divide the sample into an experimental group and a control group, use the results of the control group as the counterfactual result of the experimental group, and calculate the difference between the two results to obtain the causal effect. Compared with other methods, the most significant advantage of this method is that it can use tracking data to control the influence of unobservable variables, especially the influence of factors that do not change over time or synchronously over time, thus achieving the identification of policy effects. Under certain circumstances, there are time differences in policy implementation. For example, a policy is gradually promoted from the pilot. It constitutes the gradualness of policy implementation, and at this time, it is necessary to use the progressive double difference model to evaluate the policy effect [51].

This study intends to evaluate the effect of E-commerce development on improving the quality of agricultural development. Since the construction of NEDCs is promoted by stages and batches, it is appropriate to adopt the progressive double difference model. Under the setting of the progressive dual difference model, the national E-commerce demonstration cities after the pilot are the experimental group, and the non-national E-commerce demonstration pilot cities and the national E-commerce demonstration cities before the pilot are both the control group. In addition, we also control the time-fixed effect and the city-fixed effect when estimating the dual difference model. At the same time, the standard error is clustered at the city level to control the autocorrelation effect in cities while allowing systematic differences in the quality of agricultural development between cities.

Therefore, the empirical regression model of this study is set as follow:

$$tfp_{it}=\alpha +\beta_{1}whether_{it}+\sum \varphi_{j}x_{it}+\lambda_{t}+\mu_{i}+\varepsilon_{it}$$ (1)

In the formula (1), subscript i refers to the city, t refers to time, and tfp is the measurement of the agricultural development quality of the city. Whether is the virtual variable of whether the city is a NEDCs, which is also the core explanatory variable of this paper. *x*_*it*_ is the control variable that affects the high-quality development of agriculture and changes with city and time. *λ*_*t*_ and *μ*_*i*_ is the fixed effect of time and city respectively. *ε*_*it*_ is the residual term.

### 4.2. Variable selection

#### 4.2.1 Dependent variable: Agricultural total factor productivity and its decomposition

Based on different understandings of the connotation of high-quality development, scholars usually use two kinds of methods to measure high-quality development. One group advocates that a single "approximate" indicator should be used to measure the quality of economic development, while the other believes that the indicator system should be constructed from different dimensions to evaluate high-quality economic development comprehensively. In this paper, the agricultural total factor productivity measured by the Fare-Primont index method is used to measure cities’ high-quality agricultural development level [52]. Compared with other single indicator measures, total factor productivity has higher consistency with the connotation of high-quality economic development. Meanwhile, improving total factor productivity is also the core requirement for achieving high-quality economic development. Therefore, it can be a good measure of high-quality economic development. Compared with the index system measurement and other total factor productivity measurement methods, the Fare-Primont index method does not have the problem of subjectivity in determining the weight of agricultural total factor productivity. Since the Fare-Primont index satisfies both product completeness and transitivity, the obtained agricultural development quality index can be used to study the longitudinal change trend of high-quality development and make a horizontal comparison [53].

Referring to the existing research [26] and considering the availability of data, when the Fare-Primont index method is used to measure the agricultural total factor productivity of prefecture level cities in China, the input indicators mainly include: (1) the number of employed persons in the primary industry (ten thousand people), (2) the total power of agricultural machinery (ten thousand kilowatts), (3) the amount of chemical fertilizer applied (ten thousand tons), (4) the total sown area of crops (one thousand hectares), (5) the effective irrigation area (one thousand hectares). The output index is the added value of the primary industry (100 million yuan) at the same price in 2004. In the empirical study, in addition to taking agricultural total factor productivity (*tfp*) as the dependent variable to test the overall impact of E-commerce on the high-quality development of agriculture, agricultural total factor productivity is further decomposed into technical efficiency (*ote*), scale efficiency (*ose*) and residual mixed efficiency (*rme*). Furthermore, the decomposed indicators are taken as dependent variables to analyze the sources of e-commerce affecting the high-quality development of agriculture.

#### 4.2.2 Independent variable: Whether the city is a NEDCs

The purpose of national E-commerce demonstration city construction is to take adequate measures to promote the development of urban E-commerce and form a "demonstration effect." Therefore, compared with non-pilot cities, the national E-commerce demonstration pilot cities firmly push E-commerce development. In order to measure the development of E-commerce, we regard the pilot national E-commerce demonstration cities as cities with strong E-commerce development, with a value of 1. Cities that did not participate in the pilot and national E-commerce demonstration cities before the pilot are regarded as cities with weak E-commerce development, and the value is 0.

#### 4.2.3 Control variable

High-quality economic development in the new era is centered on the five development concepts of "innovation, coordination, green, openness, and sharing". Considering the relevance of the five development concepts and referring to the relevant studies on the influencing factors of high-quality development [35], the control variables in the empirical research mainly include: (1) human capital (*edu*), which represents variable related to innovation development. (2) agricultural scale (*agri*) and industrial structure (*pindus*), which represent variable related to coordinated development. (3) government intervention (*gov*) and financial development level (*fin*), which represent variables related to green development. (4) the level of Internet development (*pinternet*) and the degree of openness (*open*), which represent variables related to open development. (5) population size (*population*) and economic development level (*rpgdp*), which represent variables related to shared development.

The empirical research in this paper mainly involves the pilot data of NEDCs’ construction and economic and social development data of prefecture-level cities from 2004 to 2018. Among them, the pilot data of "national E-commerce demonstration cities" are manually counted according to the list of pilot cities approved by the National Development and Reform Commission and the Ministry of Commerce. The economic and social development data of prefecture-level cities are mainly from the China regional economic database of EPS data platform and the statistical yearbook of Chinese cities in the corresponding years from 2004 to 2018, supplemented by the statistical yearbooks and Statistical Bulletins of different provinces and cities in the corresponding years. All value variables were adjusted to actual values through the consumer price index in 2004. The definitions and descriptive statistics of the variables involved in the study are shown in Table 1.

**Table 1:** Definition and descriptive statistics of variables.

Variable type	Variable	Variable definition	Number	mean	standard deviation	min	max
dependent variable	*tfp*	total factor productivity	3735	0.07	0.05	0.01	0.75
	*ote*	technical efficiency	3735	0.51	0.23	0.10	1.00
	*ose*	scale efficiency	3735	0.70	0.20	0.18	1.00
	*rme*	residual mixing efficiency	3735	0.39	0.13	0.05	1.00
independent variable	*whether*	Whether it is a NEDCs (Yes = 1, no = 0)	3735	0.08	0.27	0	1
control variable	*population*	population size: logarithm of population	3735	1.25	0.72	-1.79	3.53
	*rpgdp*	Economic development level: logarithm of per capita real GDP	3734	0.19	0.72	-1.55	3.17
	*pinternet*	Internet development level: logarithm of Internet users per million people	3730	2.176	1.078	-5.109	5.292
	*agri*	Agricultural scale: logarithm of added value of primary industry	3735	4.58	0.95	-0.00	7.23
	*pindus*	Industrial structure: the proportion of added value of the secondary industry in GDP	3735	47.60	11.16	14.84	90.97
	*edu*	Human capital: logarithm of the number of college students per 10000 people	3701	4.41	1.16	-0.52	7.18
	*open*	Degree of opening up: the proportion of total import and export in GDP	3731	21.94	52.07	0.00	1066.94
	*gov*	Government intervention: ratio of fiscal expenditure to GDP	3735	17.75	12.42	4.05	234.88
	*fin*	Financial development level: ratio of balance of deposits and loans of financial institutions to GDP	3735	215.20	108.73	50.81	1353.03

### 4.3 Parallel trend test

An important prerequisite for using the double difference method to evaluate policy effectiveness is that the sample meets the essential requirement of parallel trends. That is, before the implementation of the policy, the experimental group and the control group have the same time trend. In order to intuitively investigate the dynamic difference in agricultural development quality between national E-commerce demonstration cities and non-demonstration cities, we used the method of changing the window width before and after the construction of national E-commerce demonstration cities to test the change of differences in different periods before and after the pilot. The specific regression equation is set as follows:

$$tfp_{it}=\alpha_{0}+\sum_{k\geq -5}^{5}\alpha_{k}D_{it}^{k}+\beta X_{it}+v_{t}+u_{i}+\varepsilon_{it}$$ (2)

**Fig 2:**
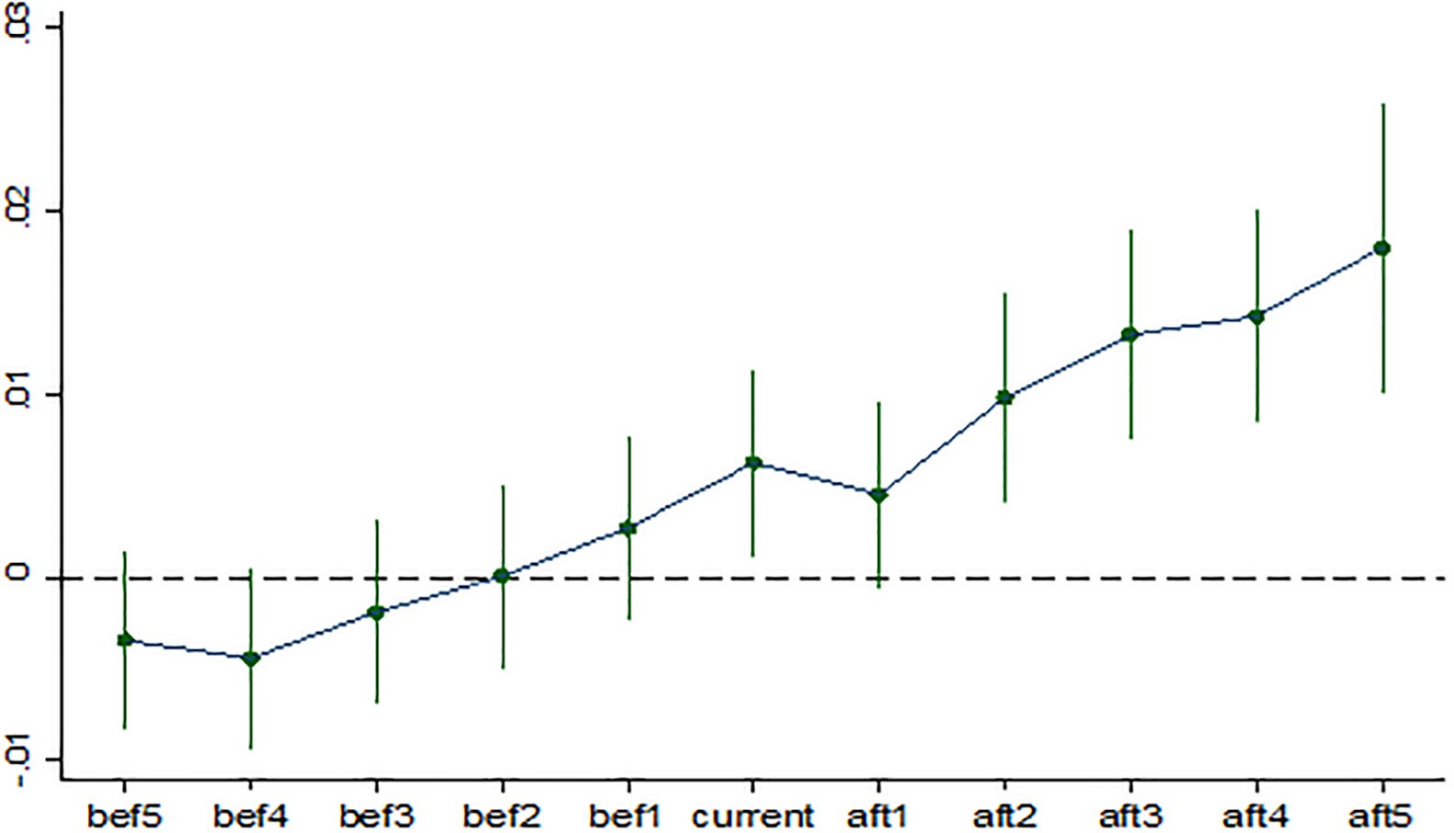
Parallel trend test results.

In formula (2), $D_{it}^{k}$ is a dummy variable representing the "event" of the construction of national E-commerce demonstration city. Specifically, *t*_*ih*_ is the specific year when city *i* carried out the construction of the national E-commerce demonstration city, *t* − *t*_*ih*_ = *k*(*k* = −5,−4,…,5) represents five years ago, four years ago,…, the current year,…, and after five years of the construction of the national E-commerce demonstration city. When *t* − *t*_*ih*_ = *k*, $D_{it}^{k}=1$, otherwise $D_{it}^{k}=0$. The parameter *α*_*k*_ reflects the impact of the national E-commerce demonstration city construction year on the high-quality development of agriculture. The estimation results of different years based on Eq (2) are shown in Fig 2.

It can be seen from Fig 2 that before the pilot construction of the national E-commerce demonstration city, the estimated values in different years were small and statistically insignificant. However, in the year and after the pilot construction of the national E-commerce demonstration city, the estimated value is immense and basically significant at the significance level of 10%. Therefore, before the pilot construction of national E-commerce demonstration cities, there was no significant difference in the quality of agricultural development between pilot cities and non-pilot cities. That is, they have similar time variation trends, and the parallelism trend hypothesis is supported. It also shows that the research object meets the primary conditions for adopting the double difference method, and the empirical model setting of this study is reasonable.

## 5. An empirical test of the impact of E-commerce development on high-quality agricultural development

### 5.1 Overall impact and its source

E-commerce, developed based on the Internet and modern information technology, has the advantages of high efficiency, low cost, real-time transparency, and transcending time and space constraints. It has dramatically improved the problems of imperfect traditional agricultural information and unimpeded sales [54] and given new vitality to the traditional agricultural production and marketing model.

From the perspective of investment, by promoting information sharing, factor flow, resource docking, and other ways, the development of E-commerce will not only force agriculture to change from the extensive development of relying on material factor input, blind production, and low efficiency in the past to the informatization, mechanization, standardization, and intensification of E-commerce trading, but also attract young labors to return home for employment and improve the cultural quality of farmers. Thus, it has a positive effect in accumulating agricultural human capital [55].

From the perspective of output, with the Internet as the carrier, E-commerce development provides opportunities for connecting producers and consumers and innovating the circulation mode of agricultural products [13]. Thus, the problem of low-price sales caused by slow sales and asymmetric information can be effectively alleviated. While increasing the sales volume, the selling price of agricultural products is guaranteed. It has promoted the growth of agricultural product sales and output in terms of "saving expenditure" and "increasing income". Moreover, E-commerce development requires the standardization, commercialization, and branding of agricultural products based on the large-scale production of agricultural products and the building of regional brands. Therefore, E-commerce development will also force agriculture to expand its scale of production and operation. Whether it is the improvement of the efficiency of input factor allocation or the increase of agricultural output caused by the expansion of production and operation scale and the growth of production and sales, all reflect the positive impact of the city’s E-commerce development on its agricultural development quality.

The estimation results in Table 2 show that: When *tfp* is the dependent variable, the coefficient estimate of *whether* is positive and significant at the significance level of 1%. This shows that the construction of national E-commerce demonstration cities has significantly improved pilot cities’ agricultural total factor productivity. This confirms that, on the whole, E-commerce development has a positive impact on the high-quality development of agriculture.

**Table 2:** Overall impact and source estimation.

Variable	(1) *tfp*	(2) *ote*	(3) *ose*	(4) *rme*
*whether*	0.0119*** (0.0014)	0.0262*** (0.0070)	0.0227*** (0.0066)	-0.0155*** (0.0044)
*population*	0.0285*** (0.0054)	0.0288 (0.0277)	-0.0609** (0.0259)	0.0580*** (0.0175)
*rpgdp*	0.0181*** (0.0030)	0.0470*** (0.0153)	0.0521*** (0.0143)	0.0030 (0.0096)
*pinternet*	-0.0015** (0.0007)	-0.0018 (0.0036)	-0.0065* (0.0034)	0.0050** (0.0023)
*agri*	0.0145*** (0.0020)	0.1827*** (0.0104)	-0.0381*** (0.0098)	-0.0031 (0.0066)
*pindus*	-0.0004*** (0.0001)	-0.0007* (0.0004)	-0.0032*** (0.0003)	0.0006*** (0.0002)
*edu*	-0.0025*** (0.0009)	-0.0040 (0.0045)	-0.0118*** (0.0042)	0.0076*** (0.0029)
*open*	-0.0000*** (0.0000)	0.0001 (0.0000)	-0.0001*** (0.0000)	0.0001*** (0.0000)
*gov*	-0.0002*** (0.0000)	-0.0007*** (0.0002)	-0.0006*** (0.0002)	0.0004*** (0.0001)
*fin*	-0.0000*** (0.0000)	-0.0001** (0.0000)	0.0000 (0.0000)	-0.0000 (0.0000)
Time fixed effect	Yes	Yes	Yes	Yes
Urban fixed effect	Yes	Yes	Yes	Yes
Number	3692	3692	3692	3692
R-squared	0.4638	0.2723	0.4609	0.7789

Note:

*, * *, * * * respectively indicate that they are significant at the significance level of 10%, 5% and 1%, and the standard error in brackets.

After decomposing total factor productivity (*tfp*) into technical efficiency (*ote*), scale efficiency (*ose*), and residual mixed efficiency (*rme*), the estimation results in Table 2 show that: When *ote* and *ose* are dependent variables, the coefficient estimates of whether are both positive and significant at the significance level of 1%. This shows that with E-commerce development, the technical efficiency and scale efficiency of agricultural development in the city have been significantly improved. When *rme* is the dependent variable, the coefficient estimate of *whether* is significantly negative at the significance level of 1%. This shows that with the development of E-commerce and the excessive inflow of capital, E-commerce will reduce of the efficiency of urban resource allocation. From the relative size of the coefficient estimates, because the positive impact of E-commerce on *ote* and *ose* is significantly higher than its negative impact on *rme*, the overall E-commerce development shows a positive impact. It can be seen that although E-commerce development will promote the improvement of technical efficiency and scale efficiency, it will also lead to the reduction of residual hybrid efficiency. The overall positive impact of E-commerce development on the high-quality development of agriculture mainly stems from its relatively more significant promotion of technical efficiency and scale efficiency.

### 5.2 Heterogeneity of impacts

As an essential form of the Internet economy, E-commerce development has the characteristics of high fixed investment and low marginal cost. In cities with large populations, the potential audience of E-commerce is also significant, which can attract more participants to connect to the E-commerce network. More E-commerce participants will not only reduce the average fixed cost borne by individuals and improve the efficiency of logistics and technology diffusion related to E-commerce development, but also promote the deepening of E-commerce network division of labor, encourage E-commerce enterprises and local governments to increase investment to improve E-commerce related supporting facilities. The deepening of labor division and the scale economy of supporting facilities construction means that with the expansion of population scale, the positive impact of E-commerce development on the high-quality development of agriculture in the city will also increase.

As a relatively new business model, E-commerce participation requires individuals to have robust Internet operation and market information mastery skills. Rural E-commerce development is influenced by various factors such as individual essential characteristics such as age and education level, family characteristics such as population size and social capital, as well as external environmental characteristics such as logistics convenience and communication infrastructure improvement [4,56]. The more developed the economy, the stronger the ability of farmers and consumers to participate in E-commerce, the better the external environment required for E-commerce development, and the fuller the role of E-commerce. Therefore, the development level of the Internet will directly determine the effectiveness of the connection between the supplier and the demander. A higher development level of the Internet will more fully stimulate the vitality of the E-commerce market and better promote improving the quality and efficiency of agriculture relying on E-commerce.

Cities with different primary development conditions, such as population size, economic development level, and Internet development level, have different "Sufficiency" of E-commerce and may impact high-quality agricultural development differently. In order to test the heterogeneous impact of E-commerce development on the high-quality development of urban agriculture under different development primary conditions, we added the cross term between the NEDCs pilot variable (*whether*) and the variables of various development primary conditions to the regression Eq (1). We used the estimated coefficient of the cross term as the discrimination of the heterogeneity of the impact of E-commerce development. The correlation and regression results are shown in Table 3.

**Table 3:** Heterogeneity test of influence.

Variable	(1) *tfp*	(2) *tfp*	(3) *tfp*
*whether*×*population*	0.0081*** (0.0019)		
*whether*×*rpgdp*		0.0267*** (0.0017)	
*whether*×*pinternet*			0.0207*** (0.0017)
control variable	Yes	Yes	Yes
Time fixed effect	Yes	Yes	Yes
Urban fixed effect	Yes	Yes	Yes
Number	3692	3692	3692
R-squared	0.4666	0.4991	0.4868

Note:

*, * *, * * * respectively indicate that they are significant at the significance level of 10%, 5% and 1%, and the standard error in brackets.

The estimation results in Table 3 show that in different regression equations, coefficient estimates of the cross term *whether×population*, *whether×rpgdp* and *whether×pinternet* are all positive and significant at the significance level of 1%. This confirms that the impact of E-commerce development on the high-quality development of agriculture will vary according to the primary conditions of urban development. The larger the population, the higher the level of economic development, and the higher the level of Internet development, the more significant the positive impact of E-commerce development on the high-quality development of agriculture.

### 5.3 Analysis of dynamic impact mechanism

The positive impact of e-commerce development policies on high-quality agricultural development is reflected in the improvement of technological innovation capabilities and economies of scale, as well as the optimization of resource allocation efficiency. From the perspective of technological innovation, e-commerce, as a new business model based on the Internet, has put forward higher requirements for the standardization level, quality and safety, and yield stability of agricultural products. This will not only lead to improvements in deep processing and packaging, as well as innovation in agricultural product technology, but also accelerate the updating of advanced agricultural production technologies such as large-scale planting and breeding, sensor monitoring, etc. Ultimately, it enhanced the innovation capability of agricultural technology. From the perspective of economies of scale, the development of e-commerce has broken down industry barriers, expanded and extended the agricultural product industry chain, and attracted the entry of non-agricultural capital. The development of e-commerce has also driven the development of upstream and downstream industries, such as logistics, payment, information technology services, etc., and then promote enterprises to expand production scale along the industrial chain and improve production efficiency. It promotes the scale production of agriculture and improves the scale economy effect of agricultural production. From the perspective of resource allocation, the excellent income prospects of agricultural e-commerce attract a large amount of capital into the agricultural field, promote the adjustment of the ratio of capital and resources, and optimize the efficiency of resource allocation. When capital inflows exceed the desired scale required for agricultural resource endowments, the marginal return on capital declines. Therefore, the impact of e-commerce development on the efficiency of agricultural resource allocation will depend on the relative abundance of capital and agricultural resources.

The inhibiting effect of e-commerce development policies on the high-quality development of agriculture is reflected in: with the gradual release of the potential of e-commerce technology upgrades, agricultural technology will not be significantly improved because of the development of e-commerce. The impact of e-commerce on agricultural scale economies is limited by the most extensive range, and when the optimal range is reached, it will lead to the decline of agricultural scale economies. When the influence of e-commerce on agricultural resource endowment exceeds the ideal proportion, the marginal return on capital will decline.

Therefore, the impact of E-commerce development on agriculture’s high-quality development may show dynamic sustainability. In terms of technological innovation, economies of scale and resource allocation, the impact of E-commerce includes not only factors that are gradually strengthened, but also elements that cause attenuation. Its impact on different sources of high-quality development results from interacting factors in different directions.

In order to investigate the temporal change trend of the impact of urban E-commerce development on the high-quality development of agriculture, we further added the cross term of the NEDCs pilot variable whether and the virtual variable of the pilot time to the regression Eq (1), and discriminated the dynamic impact of E-commerce development on the high-quality development of agriculture according to the coefficient estimate of the cross term (show in Table 4). Furthermore, since the pilot’s second year, the estimated value of the coefficient has been increasing. This shows that, on the whole, the development of E-commerce has a continuous impact on the high-quality development of agriculture. The role of E-commerce is gradually playing, and its positive impact on the high-quality development of agriculture is gradually increasing.

**Table 4:** Dynamic test of influence.

year	(1) *tfp*	(2) *ote*	(3) *ose*	(4) *rme*
Pilot year	0.0086*** (0.0023)	0.0158 (0.0117)	0.0110 (0.0109)	-0.0074 (0.0074)
The first year of pilot	0.0066*** (0.0023)	0.0215* (0.0117)	0.0114 (0.0109)	-0.0089 (0.0074)
The second year of pilot	0.0121*** (0.0026)	0.0342*** (0.0132)	0.0207* (0.0124)	-0.0118 (0.0083)
The third year of pilot	0.0157*** (0.0026)	0.0345*** (0.0133)	0.0336*** (0.0125)	-0.0215** (0.0084)
The fouth year of pilot	0.0166*** (0.0026)	0.0339** (0.0135)	0.0299** (0.0126)	-0.0215** (0.0085)
The fifth year of pilot	0.0206*** (0.0038)	0.0254 (0.0196)	0.0635*** (0.0183)	-0.0487*** (0.0123)
The sixth year of pilot	0.0240*** (0.0038)	0.0294 (0.0197)	0.0594*** (0.0184)	-0.0395*** (0.0124)
control variable	Yes	Yes	Yes	Yes
Time fixed effect	Yes	Yes	Yes	Yes
Urban fixed effect	Yes	Yes	Yes	Yes
Number	3692	3692	3692	3692
R-squared	0.4683	0.2728	0.4629	0.7799

Note:

*, * *, * * * respectively indicate that they are significant at the significance level of 10%, 5% and 1%, and the standard error in brackets.

With *ote* as the dependent variable, the coefficient estimate of the cross term between *whether* and the pilot time is significantly positive only from the first year to the fourth year of the pilot, and the magnitude and significance level of the coefficient estimate increase first and then decrease during this period. It shows that the impact of E-commerce development on agricultural technology innovation has a short time lag and a particular time window. The time lag period and window period are about 1 year and 4 years respectively, and the positive impact increases first and then decreases in the window period.

With *ose* as the dependent variable, the coefficient estimates of the cross term of *whether* and the pilot time is significantly positive from the second year, and then the size and significance level of the coefficient estimate gradually increase until it reaches the maximum in the fifth year of the pilot and then starts to decrease. This means that the impact of E-commerce development on agricultural economies of scale has a relatively longer time lag (technological innovation), and after the time lag period, the positive impact of E-commerce also shows a trend of first increasing and then decreasing.

Taking *rme* as the dependent variable, the coefficient estimates of the cross term between *whether* and the pilot time are significantly negative from the third year, and then the absolute value and significance level of the coefficient estimate gradually increase, reaching the maximum in the fifth year of the pilot, and then decreasing. This confirms that although E-commerce development harms agricultural resource allocation, there is a long time lag in its impact, and after the time lag period, the negative impact of E-commerce development first increases and then decreases.

To sum up, E-commerce development’s overall positive impact on agriculture’s high-quality development gradually increases over time. However, its impact on agricultural technology innovation, economies of scale, and resource allocation has different time lags. After the time lag period, E-commerce development has a continuous positive impact on agricultural technology innovation and scale economy, which increases first and then decreases, while it has a continuous negative impact on agricultural resource allocation, which increases first and then decreases. It can be inferred from this that the E-commerce development has gradually increased the positive impact on the high-quality development of agriculture. At the beginning, it was mainly due to improved agricultural technology innovation and scale economy caused by E-commerce, and at the later stage, it was mainly due to the reduction of its negative impact on agricultural resource allocation.

### 5.4 Robustness of estimation results

#### 5.4.1 Eliminate the impact of disruptive policies

During the pilot construction period of NEDCs, many policies and measures closely related to high-quality economic development are also being promoted simultaneously. If national E-commerce demonstration cities also implement these policies and measures simultaneously, it may have a confusing impact. Then we will not be able to distinguish whether the improvement of agricultural development quality in pilot cities comes from the effect of E-commerce or other policies and measures related to high-quality economic development. So we have taken the national low-carbon pilot cities [57], carbon emission trading pilot cities [58], innovative city pilot cities [59], high-speed rail opening [60], and other policies and measures that have been proven to have a significant positive impact on economic quality as disruptive policies. In regression Eq (1), dummy variables representing these policies are added respectively to examine the changes in the impact of E-commerce development on high-quality agricultural development after controlling the impact of different disruptive policies. On this basis, the robustness of the estimation results is judged. The regression results after controlling the impact of relevant disruptive policies are shown in Table 5.

**Table 5:** Estimation of controlling the impact of different disruptive policies.

Control variable	(1) *tfp*	(2) *tfp*	(3) *tfp*	(4) *tfp*
*whether*	0.0104*** (0.0014)	0.0103*** (0.0013)	0.0092*** (0.0014)	0.0113*** (0.0014)
National low carbon pilot cities	0.0084*** (0.0011)			
Pilot cities for carbon emission trading		0.0196*** (0.0014)		
Innovative city pilot			0.0122*** (0.0015)	
High speed railway opened				0.0047*** (0.0009)
control variable	Yes	Yes	Yes	Yes
Time fixed effect	Yes	Yes	Yes	Yes
Urban fixed effect	Yes	Yes	Yes	Yes
Number	3692	3692	3692	3692
R-squared	0.4730	0.4925	0.4741	0.4678

Note:

*, * *, * * * respectively indicate that they are significant at the significance level of 10%, 5% and 1%, and the standard error in brackets.

Table 5 shows that in each regression, the estimated coefficients of different interfering policy dummy variables are positive, and significant at the 1% significance level. It shows that these policies really have a positive impact on the high-quality agricultural development of the city. Even though the estimated coefficient of the pilot variable NEDCs in different regressions is not only significantly positive, but also unchanged. It shows that after controlling the impact of disruptive policies, E-commerce development still has a significant positive impact on high-quality agricultural development, which confirms the robustness and reliability of the estimation results.

#### 5.4.2 Estimation of different estimation methods

This paper aims to assess the impact of the pilot cities of the experimental group participating in the NEDCs on the quality of their agricultural development. Thus, it reveals the causal relationship between E-commerce development and high-quality agricultural development. In reality, whether a specific city is selected as a NEDCs pilot is largely non-random, which leads to sample selection bias. In addition, the difference between the experimental group and the control group in the quality of urban agricultural development can also be derived from other unpredictable factors that over time, and the potential missing variables will also lead to inconsistent estimation results. Considering the possible impact of selective and missed variable bias on the estimation results, this study further uses the PSM-DID method [3,58] and instrumental variable method [61] to make robust estimations. The specific estimation results are shown in Table 6.

**Table 6:** PSM-DID estimation and tool variable estimation.

Variable	(1) PSM-DID estimation	(2) Tool variable estimation
*whether*	0.0092*** (0.0026)	0.0163*** (0.0041)
control variable	Yes	Yes
Time fixed effect	Yes	Yes
Urban fixed effect	Yes	Yes
Anderson canon. corr. LM statistic		404.629***
Cragg-Donald Wald F statistic		29.932 [21.18]
Sargan statistic		94.209***
Number	832	3735

Note:

*, * *, * * * respectively indicate that they are significant at the significance level of 10%, 5% and 1%, and the standard error in brackets.

The PSM-DID estimation in Table 6 shows that although the sample size has decreased significantly after matching, the estimated value of the coefficient of the pilot variable *whether* has hardly changed and is still significantly positive at the 1% significance level. In the estimation of tool variables, the selected tool variables passed the corresponding identification test, reflecting the rationality of the tool variables. Then, the size and significance level of the estimated value of the pilot variable, the coefficient estimate of *whether* still has not changed much compared with the benchmark regression. In general, though PSM-DID estimation and tool variable estimation, the coefficient estimate of *whether* is not only significantly optimistic at the 1% significance level but also maintain a high stability. This again confirms the robustness of the estimated results in the benchmark as mentioned above regression.

#### 5.4.3 Placebo test

The principle of the placebo test is that if the high-quality agricultural development is caused by other factors rather than the E-commerce development of the pilot cities, then assuming that the pilot cities have carried out the pilot, significant results will be obtained. On the contrary, the improvement of agricultural development quality comes from the role of urban E-commerce development. Therefore, this paper adopts the counterfactual method to conduct the placebo-controlled test at the same time. Namely, the randomly selected city (year) is set as the national E-commerce demonstration pilot city (year), which is used as the basis for the grouping and staging of the pilot "experiment" to perform double difference estimation. The robustness of the benchmark regression is judged according to the size (significance) and distribution of the estimates of the differential term.

**Fig 3:**
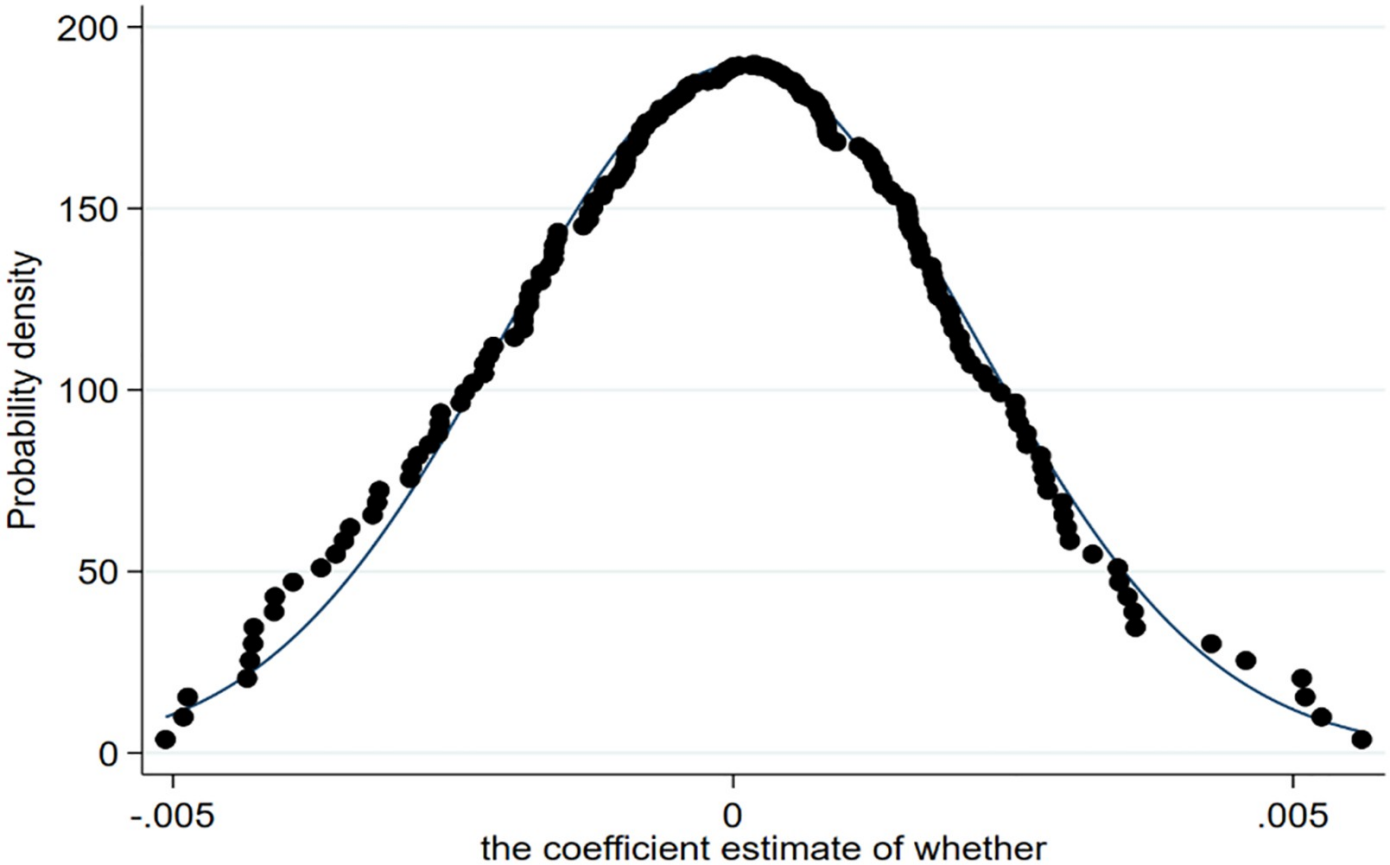
Estimation of 200 random samples.

Referring to La Ferrara et al. [62], the specific approach of this study is: in all samples, 25% of the samples are randomly selected as the samples impacted by the pilot "events", and the sample cities are selected as the pilot cities. The time is regarded as the starting time of the pilot project. On this basis, the double difference sub-item "who" is reconstructed according to the empirical thinking of the double difference model. Then, a double difference estimation of panel data is carried out, and the impact of E-commerce development on high-quality agricultural development in this placebo test is judged by the coefficient estimate of *whether*. To ensure the randomness of grouping and enhance the persuasiveness of the results, repeat the above process 200 times. That is, 200 random grouping and corresponding double difference estimations. The distribution of the estimated value of the 200 sampling “who” coefficient is shown in Fig 3. The estimation results in Fig 3 show that under 200 random grouping, the estimated value of the coefficient of the randomly selected pilot variable *whether* is a normal distribution close to 0. It is not only far less than the results of the benchmark regression of this study, but also not significant at the 10% significance level. This shows that the significant positive impact of E-commerce development in the benchmark regression is not driven by other unobserved factors, which also proves the robustness of the benchmark regression results.

## 6. Conclusion and discussion

### 6.1 Conclusions

With the increasingly fierce competition in the urban E-commerce market, E-commerce to the countryside has become an inevitable trend of China’s E-commerce development. At the moment of actively promoting the "rural revitalization", how to seize the strategic opportunities brought by the development of rural E-commerce to promote the high-quality development of agriculture has become a rural area, consideration for practitioners in the field of "agriculture, rural areas, and farmers" when making agricultural related decisions. In order to clarify the relationship between E-commerce and high-quality agricultural development, this study regards the NEDCs pilot as a quasi-natural experiment of urban E-commerce development. Based on the E-commerce pilot and economic and social development data of prefecture-level cities nationwide from 2004 to 2018, this study empirically tested the impact of E-commerce development on high-quality agricultural development in China and its sources, analyzed the impact’s heterogeneity and dynamics and verified the impact mechanism. The research results show that:

Overall, as an E-commerce development policy, NEDC’s policy positively impacts on China’s high-quality agricultural development. Regarding its source, the positive impact of NEDC policy on high-quality agricultural development stems from its combined effect on technological innovation, economies of scale, and resource allocation. Specifically, NEDC policy has a significant role in promoting technological innovation and economies of scale, while it significantly negatively impacts resource allocation. However, its promoting effect on the former two is higher than its adverse effect on the latter.

From the perspective of heterogeneity and dynamics of impacts, the impact of NEDCs policy on high-quality agricultural development varies according to the primary conditions of urban development. The larger the population size of a city, the higher the level of economic development or Internet development, and the more significant the positive impact of NEDC policy on its high-quality agricultural development. Overall, the impact of NEDC policy on high-quality agricultural development shows dynamic sustainability, and its impact intensity first increases and then decreases. From the source, the impact of NEDC policy on technological innovation, economies of scale, and resource allocation shows different time lags. After the time lag, the impact intensity shows a trend of increasing first and then decreasing.

Overall, the empirical research in this paper confirms that the E-commerce development policy has a significant, positive, and sustainable impact on high-quality agricultural development. This not only dramatically enriches the literature on the impact of e-commerce [8–11] and the development of agricultural e-commerce [21,43], providing empirical support for the rationality and effectiveness of various forms of e-commerce to assist agriculture, but also provides empirical evidence for local governments to use E-commerce to enable high-quality agricultural development. In addition, this study also found that for cities with good primary conditions for development, E-commerce development has a more significant positive impact on high-quality agricultural development. This not only indicates that cities with good development foundations can more effectively use the efficiency improvement brought by E-commerce to promote their own development but also means that in the process of boosting high-quality agricultural development with E-commerce, local governments should focus on the construction of supporting facilities related to E-commerce development, so as to fully stimulate the potential of E-commerce to improve the quality of economic development. It is worth noting that, as the development of E-commerce may have a continuous adverse impact on the allocation of agricultural resources, the matching between the development level of E-commerce and the endowment of agricultural resources should also be paid attention to during the inflow of relevant capital and resources to the agricultural E-commerce field. Only by promoting the development of agriculture-related E-commerce based on desirable agricultural resource endowment can the role and effectiveness of E-commerce be entirely played.

The main contributions of this article are in three aspects. Firstly, the agricultural total factor productivity index measured by the Fare Primont index method is used to measure the high-quality agricultural development in cities. Compared with the multi-indicator evaluation index system measurement and traditional total factor productivity measurement methods, the Fare Primont index method can not only effectively reflect the multidimensional characteristics of high-quality agricultural development, but also can be used for vertical and horizontal comparison of high-quality agricultural development. Secondly, taking the construction of "National E-commerce Demonstration Cities" as a quasi-natural experiment to promote the development of e-commerce in cities, using a double difference model to test the impact of e-commerce on high-quality agricultural development can effectively reduce endogeneity and more reliably identify the high-quality development effects of e-commerce development promotion policies. Thirdly, it not only examined the overall impact of e-commerce on high-quality agricultural development, but also examined the heterogeneity and dynamics of e-commerce’s impact on high-quality agricultural development, thus comprehensively evaluating the policy effects of promoting e-commerce development.

### 6.2 Recommendation

#### 6.2.1 Strengthen infrastructure construction support for agricultural E-commerce development

NEDCs often have a good network foundation for implementation. The construction of agricultural network infrastructure plays a crucial and decisive role in developing agricultural E-commerce. The government should increase the infrastructure construction of the agricultural Internet of Things and the Internet and elevate the construction of agricultural information infrastructure to an equally important position as traditional infrastructure such as water conservancy, electricity, and transportation. It is necessary to improve the layout of infrastructure networks and promote the construction of agricultural information industry clusters. First, strengthen the construction of rural broadband access ports and network communication base stations, constantly improve bandwidth and upgrade network speed, and promote information services to go deep into rural grassroots. Second, accelerate the construction of communication network infrastructure represented by 5G, the Internet of Things, and the satellite Internet, and build a new generation of secure, mobile, and high-speed agricultural information infrastructure.

#### 6.2.2 Increase investment support for agricultural technology innovation

Empirical research shows that NEDC’s policy promotes the high-quality development of agriculture mainly through technological innovation and economies of scale. On the one hand, it is necessary to increase investment in agricultural science and technology to promote the development of the agricultural digital technology industry, organically integrate a new generation of information technology with agricultural equipment manufacturing, and improve the level of agricultural equipment and the quality and efficiency of agricultural machinery operations. On the other hand, we should build an information exchange platform among relevant subjects such as farmers, enterprises, scientific research institutions, and governments to achieve multi-level real-time feedback and exchange of agricultural information. At the technical level, it is necessary to vigorously develop the "new infrastructure" of digital agriculture represented by agricultural data centers and build an agricultural big data cloud sharing platform, an intelligent agricultural monitoring platform, and a traceability platform for agricultural products.

#### 6.2.3 Enhancing support for the cultivation of new farmers

The practical implementation of the NEDCs policy depends on people. Similarly, the success of agricultural E-commerce development depends on farmers’ application of E-commerce technology. Therefore, it is necessary to improve the ability of farmers to obtain agricultural information and cultivate a new type of modern farmers who understand agriculture, are good at management, can manage, and have Internet thinking. It is necessary to strengthen the cultivation of new types of farmers and incorporate the training of new types of farmers into the vocational education plan and the economic and social development plan. Through targeted training in information knowledge, agricultural production technology, and modern management, farmers are helped to improve their ability to obtain agricultural information by digital means and their ability to operate and manage.

### 6.3 Research limitations and future research directions

This study has the following limitations. First, the high-quality development of agriculture involves many aspects of agricultural development. Suppose only the agricultural total factor productivity is taken as the measurement of the high-quality development of agriculture. In that case, there may be a problem in that the accuracy of the measurement results could be higher due to the narrow measurement range. Secondly, this study only examined the impact of general e-commerce development promotion policies on high-quality agricultural development. As this policy does not target the promotion of agricultural development, the conclusions drawn may not apply to policies related to the development of agricultural e-commerce. Thirdly, although we discussed in the analysis the possible mechanism of e-commerce affecting the high-quality development of agriculture, it was not tested in the empirical study due to data limitations. Therefore, in the future, based on more detailed measurements of high-quality agricultural development and more targeted data on agricultural e-commerce development policies, a more detailed examination of the impact and mechanism of e-commerce on high-quality agricultural development can be conducted to promote the improvement of related research further.
